# Longitudinal analysis of the Non-Motor Symptoms Scale in Parkinson's Disease (NMSS): An exploratory network analysis approach

**DOI:** 10.3389/fneur.2023.972210

**Published:** 2023-02-14

**Authors:** Konstantin G. Heimrich, Aline Schönenberg, Hannah M. Mühlhammer, Sarah Mendorf, Diego Santos-García, Tino Prell

**Affiliations:** ^1^Department of Neurology, Jena University Hospital, Jena, Germany; ^2^Department of Geriatrics, Halle University Hospital, Halle, Germany; ^3^Complejo Hospitalario Universitario de A Coruña (CHUAC), A Coruña, Spain

**Keywords:** Parkinson's disease, non-motor symptoms, depression, dysautonomia, symptom assessment, scales, network analysis

## Abstract

**Introduction:**

Parkinson's disease (PD) is a multisystem neurodegenerative disorder characterized by motor and non-motor symptoms. In particular, non-motor symptoms have become increasingly relevant to disease progression. This study aimed to reveal which non-motor symptoms have the highest impact on the complex interacting system of various non-motor symptoms and to determine the progression of these interactions over time.

**Methods:**

We performed exploratory network analyses of 499 patients with PD from the Cohort of Patients with Parkinson's Disease in Spain study, who had Non-Motor Symptoms Scale in Parkinson's Disease ratings obtained at baseline and a 2-year follow-up. Patients were aged between 30 and 75 years and had no dementia. The strength centrality measures were determined using the extended Bayesian information criterion and the least absolute shrinkage and selection operator. A network comparison test was conducted for the longitudinal analyses.

**Results:**

Our study revealed that the depressive symptoms *anhedonia* and *feeling sad* had the strongest impact on the overall pattern of non-motor symptoms in PD. Although several non-motor symptoms increase in intensity over time, their complex interacting networks remain stable.

**Conclusion:**

Our results suggest that anhedonia and feeling sad are influential non-motor symptoms in the network and, thus, are promising targets for interventions as they are closely linked to other non-motor symptoms.

## 1. Introduction

Parkinson's disease (PD) is a progressive multisystem neurodegenerative disorder characterized by motor and non-motor symptoms (1). In particular, non-motor symptoms dominate the clinical picture of advanced PD and negatively impact patients' quality of life (2, 3). Therefore, the early identification and appropriate management of non-motor symptoms are important for maintaining wellbeing. The Non-Motor Symptoms Scale in Parkinson's Disease (4) was developed as a comprehensive assessment of different non-motor symptoms in PD (5). It comprises 30 items, each of which describes different non-motor symptoms. The NMSS is commonly used to detect non-motor symptoms in patients with PD and has been used as a clinical outcome measure of non-motor symptoms in many original research studies (6). However, data on longitudinal changes in NMSS are rare. A previous study revealed that the global NMSS burden increases over time (7). However, in addition to the progression of the total score, it is unclear whether the progression of a particular non-motor symptom may cause an increase in other symptoms. Considering the many non-motor symptoms that have been identified, it is crucial to understand how they are linked and whether certain non-motor symptoms are particularly influential in the sense that they in- or decrease other non-motor symptoms. This information is necessary to develop tailored interventions for patients with PD. Nevertheless, it has not been clarified which non-motor symptoms have the highest impact when considering the overall pattern of linkages between all non-motor symptoms. Network analysis is a suitable tool to address this question. Therefore, the present study aimed to reveal which symptoms of the NMSS had the highest impact on the complex interacting system of all symptoms. In a subsequent step, we aimed to clarify whether this network and the interactive pattern between non-motor symptoms changes over time.

## 2. Methods

### 2.1. Study design

Data were extracted from the COhort of Patients with Parkinson's DIsease in Spain (COPPADIS) study, a national, multicenter, non-interventional, longitudinal study (8). PD patients without dementia, aged between 30 and 75 years, were initially recruited from 35 centers in Spain from January 2016 to November 2017. Detailed information on the study design, content, and exclusion criteria is provided in the COPPADIS study protocol (8).

### 2.2. Participants

In total, the COPPADIS cohort included 694 PD patients at the baseline evaluation, of whom 690 completely filled out the NMSS. In this study, we focused on patients with PD who completed the NMSS both at the baseline evaluation and the 2-year follow-up, resulting in a sample of 499 patients.

### 2.3. Variables

To assess the non-motor symptoms, the NMSS was collected at baseline and the 2-year follow-up. The NMSS comprises 30 items, each of which describes a different non-motor symptom. Symptoms refer to the month prior to assessment. The score for each item is calculated by multiplying the severity (0 = none; 1 = mild; 2 = moderate; 3 = severe) and frequency (1 = rarely; 2 = often; 3 = frequent; 4 = very frequent), ranging from 0 to 12 points per item. The total NMSS score ranges from 0 to 360 points. The items are commonly grouped into nine different domains: cardiovascular (domain 1; items 1 and 2), sleep/fatigue (domain 2; items 3, 4, 5, and 6), mood/cognition (domain 3; items 7, 8, 9, 10, 11, and 12), perceptual problems (domain 4; items 13, 14, and 15), attention/memory (domain 5; items 16, 17, and 18), gastrointestinal tract (domain 6; items 19, 20, and 21), urinary (domain 7; items 22, 23, and 24), sexual function (domain 8; items 25 and 26), and miscellaneous (domain 9; items 27, 28, 29, and 30) (5).

In addition, the following variables were extracted at baseline and the two-year follow-up: patient age, sex, Hoehn and Yahr stage (9), Unified Parkinson's Disease Rating Scale (UPDRS) parts III and IV (10), Mini-Mental State Examination (MMSE) (11), and revised version of the Beck Depression Inventory (BDI-II) (12).

### 2.4. Statistical analyses

For descriptive statistics, data were checked for normality using the Shapiro–Wilk test, which revealed non-normal distributions for most variables. Thus, the results are reported as numbers and percentages for categorical variables, and median and interquartile range (IQR) for continuous variables. For group comparisons, the paired Wilcoxon signed-rank test was used to examine differences between the two time-points. The effect sizes of the group differences are given by the correlation coefficient r = Z/$\sqrt{N}$. Correlations can be considered low (|r| = 0.1), moderate (|r| = 0.3), or strong (|r| = 0.5) (13). The level of statistical significance for all tests was set at *p* < 0.05 (two-tailed).

Data quality was considered acceptable if >95% of the NMSS total score was fully computable. In addition, floor and ceiling effects were calculated, and a maximum of 15% was considered satisfactory (14). Internal consistency was determined using Cronbach's alpha coefficient with a minimum criterion value of 0.7 (15). Item homogeneity was assessed as the mean of the inter-item correlation coefficients with a minimum criterion value of ≥0.3 suggesting moderate correlations (13), and corrected item-total correlations with a minimum criterion value of 0.4 (16).

Exploratory network analyses based on partial correlation were conducted to explore the associations between the 30 items of the NMSS at baseline and the 2-year follow-up. In this network approach, the individual non-motor symptoms are considered as a complex interacting system. Thus, the overall pattern of linkages between the non-motor symptoms is examined to understand the interactions, rather than looking at separate correlations. However, to prevent overfitting and ensure replicability of the network structures, a regularization technique is frequently used to limit the number of spurious relationships between items (17). In this study, the network characteristics and structure of NMSS were assessed using the extended Bayesian information criterion (EBIC) (18, 19) with the least absolute shrinkage and selection operator (LASSO). To ensure a more sensitive and specific network analysis, the tuning parameter of EBICglasso was set to 0.5 (20). To achieve a normal distribution of our data, a non-paranormal transformation of non-normally distributed data was performed (npn). The individual NMSS items are presented as nodes in the network. They are positioned using the Fruchterman–Reingold algorithm based on the strength of the connections between nodes using pseudo-random numbers (21). The partial correlations between the nodes are displayed by so-called edges. The thickness of the edge corresponds to the intensity of the correlation. To assess the respective influence of a node and its connections to other nodes, several centrality measures are available. In this analysis, we used strength as a centrality measure, as this is considered the most appropriate for our type of data (22). Strength was determined using normalized values. The strength of a node refers to the sum of the absolute edge weights connected to that node (23) and, accordingly, describes the direct connections of one non-motor symptom to the other non-motor symptoms (23–25). Clinically, a node with high strength may represent an important feature or a possible therapeutic target, as a change in the value of this node can rapidly affect other nodes within the network. In addition to the primarily performed network analyses on item level of the NMSS, we conducted network analyses on domain level to reveal possible differences.

Network stability was estimated *via* a case-dropping bootstrap (number of bootstraps = 1,000) and reported using the correlation stability (CS) coefficient. The CS coefficient quantifies the proportion of cases that can be dropped to retain a correlation with the original strength of at least 0.7 in at least 95% of samples (25). The CS coefficient must generally be above 0.25, and preferably above 0.5 (25). The accuracy of the networks was estimated using non-parametric bootstrapping procedures to assess edge weight stability, with narrower 95% confidence intervals indicating more trustworthy networks (25). In addition, bootstrapped difference tests were conducted to determine whether the centrality measures of a node in the network were significantly different from each other node (25).

Moreover, we aimed to assess differences between the network of baseline data and follow-up data. Therefore, a network comparison test based on a permutation test (*n* = 1,000) was performed to assess network structure invariance, global strength invariance, and edge strength invariance (26). Thereby, network structure invariance refers to the maximum difference in pairwise edges between two networks, global strength invariance refers to the difference in the weighted absolute sum of all edges between two networks, and edge strength invariance refers to the difference in specific edge weights between two networks.

SPSS (IBM SPSS Statistics, RRID: SCR_016479, version 27), R (version 4.2.1), and JASP (JASP, RRID: SCR_015823, version 0.15) were used for statistical analyses.

## 3. Results

### 3.1. Descriptive analysis

Of the 499 patients with PD, 294 (58.9%) were male and 205 (41.1%) were female. At baseline, the median age of the patients was 64 years (IQR = 57–69 years), with a median disease duration of 5 years (IQR = 2–8 years). Most patients presented a disease stage with bilateral involvement (Hoehn and Yahr stage ≥ 2), moderate motor impairment (median UPDRS III: 20 points, IQR = 13.5–29), and at least one motor complication (median UPDRS IV, 1 point; IQR, 0–3). Most patients had neither cognitive impairment (median MMSE, 30 points; IQR, 29–30) nor relevant depressive symptoms (median BDI-II: 7 points, IQR = 3–12). According to the NMSS, patients reported non-motor symptoms with a median total score of 34 points (IQR, 19–59). Descriptive statistics of the study population at baseline and at the 2-year follow-up, including the items of the NMSS and group comparisons, are shown in Table 1. After 2 years, patients had a higher Hoehn and Yahr stage (*p* < 0.001; r = 0.286), higher UPDRS III (*p* < 0.001; r = 0.318), higher UPDRS IV (*p* < 0.001; r = 0.272), and worse MMSE scores (*p* < 0.001; r = 0.339). The NMSS total score was furthermore found to increase over time (*p* < 0.001; r = 0.270). In particular, item 4 (*fatigue*) worsened over the two-year period (*p* < 0.001; r = 0.226); however, the effect sizes were low (Table 1).

**Table 1:** Descriptive statistics of the study population and the NMSS.

	**Baseline**	**Two-year follow-up**	**Z**	***p***	**r**
*N*	499	499			
**Sex**					
Male	294 (58.9)	294 (58.9)	/	/	/
Female	205 (41.1)	205 (41.1)	/	/	/
Age (years)	64 (57–69)	66 (59–71)	/	/	/
Disease duration	5 (2–8)	7 (4–10)	/	/	/
HY off	2 (2–2)	2 (2–2)	−5.973	< 0.001^*^	0.286
UPDRS III off	20 (13.5–29)	24 (16–32)	−6.644	< 0.001^*^	0.318
UPDRS IV off	1 (0–3)	2 (0–4)	−5.956	< 0.001^*^	0.272
MMSE	30 (29–30)	29 (28–30)	−7.448	< 0.001^*^	0.339
BDI–II	7 (3–12)	7 (3–13)	−0.845	0.398	0.038
NMSS, total score	34 (19–59)	43 (22–71)	−6.040	< 0.001^*^	0.270
Cardiovascular (domain 1)	0 (0–1)	0 (0–2)	−3.397	0.001^*^	0.152
1. Light headedness	0 (0–1)	0 (0–2)	−3.457	< 0.001^*^	0.155
2. Fainting	0 (0–0)	0 (0–0)	−1.165	0.244	0.052
Sleep/fatique (domain 2)	6 (2–12)	7 (3–14)	−3.632	< 0.001^*^	0.163
3. Daytime sleepiness	1 (0–3)	1 (0–4)	−2.936	0.003^*^	0.131
4. Fatigue	1 (0–4)	2 (0–6)	−5.059	< 0.001^*^	0.226
5. Sleep initiation	0 (0–2)	0 (0–3)	−1.843	0.065	0.083
6. Restless legs	0 (0–2)	0 (0–2)	−0.030	0.976	0.001
Mood/cognition (domain 3)	3 (0–11)	4 (0–14)	−3.139	0.002^*^	0.141
7. Loss of interest	0 (0–1)	0 (0–1)	−2.958	0.003^*^	0.132
8. Lack of motivation	0 (0–1)	0 (0–2)	−2.837	0.005^*^	0.127
9. Feeling nervous	0 (0–2)	0 (0–3)	−1.089	0.276	0.049
10. Feeling sad	0 (0–2)	1 (0–3)	−2.473	0.013^*^	0.111
11. Flat mood	0 (0–1)	0 (0–2)	−2.165	0.030^*^	0.097
12. Anhedonia	0 (0–2)	0 (0–2)	−1.493	0.135	0.067
Perceptual problems (domain 4)	0 (0–0)	0 (0–1)	−5.190	< 0.001^*^	0.232
13. Hallucinations	0 (0–0)	0 (0–0)	−3.866	< 0.001^*^	0.173
14. Delusions	0 (0–0)	0 (0–0)	−3.773	< 0.001^*^	0.169
15. Diplopia	0 (0–0)	0 (0–0)	−3.120	0.002^*^	0.140
Attention/memory (domain 5)	2 (0–4)	2 (0–7)	−4.128	< 0.001^*^	0.185
16. Concentration	0 (0–2)	0 (0–3)	−2.595	0.009^*^	0.116
17. Forgetfulness	0 (0–2)	0 (0–2)	−3.548	< 0.001^*^	0.159
18. Forget to do things	0 (0–1)	0 (0–1)	−3.004	0.003^*^	0.134
Gastrointestinal tract (domain 6)	1 (0–5)	2 (1–7)	−5.163	< 0.001^*^	0.231
19. Sialorrhea	0 (0–1)	0 (0–2)	−3.294	0.001^*^	0.147
20. Dysphagia	0 (0–0)	0 (0–1)	−2.707	0.007^*^	0.121
21. Constipation	0 (0–2)	0 (0–4)	−4.127	< 0.001^*^	0.185
Urinary (domain 7)	6 (1–12)	6 (2–13)	−3.419	0.001^*^	0.153
22. Urgency	1 (0–4)	2 (0–6)	−2.827	0.005^*^	0.127
23. Frequency	1 (0–4)	1 (0–4)	−1.378	0.168	0.062
24. Nocturia	1 (0–4)	2 (0–4)	−2.808	0.005^*^	0.126
Sexual function (domain 8)	1 (0–8)	4 (0–8)	−3.000	0.003^*^	0.134
25. Interest	0 (0–4)	0 (0–4)	−2.262	0.024^*^	0.101
26. Problems having sex	0 (0–4)	0 (0–4)	−3.021	0.003^*^	0.135
Miscellaneous (domain 9)	5 (1–12)	6 (1–12)	−2.921	0.003^*^	0.131
27. Pain	0 (0–1)	0 (0–2)	−2.701	0.007^*^	0.121
28. Taste/smell	2 (0–6)	2 (0–6)	−1.103	0.270	0.049
29. Weight change	0 (0–0)	0 (0–0)	−0.899	0.369	0.040
30. Hyperhidrosis	0 (0–1)	0 (0–2)	−2.312	0.021^*^	0.103

The number of participants is given in absolute values; other values are given as the medians and interquartile ranges; categorical parameters are given as absolute values and percentages. For group comparisons, the paired Wilcoxon signed-rank test was used to examine the differences over time. BDI-II, revised version of the Beck Depression Inventory; HY, Hoehn and Yahr stage; MMSE, Mini-Mental State Examination; N, number of participants; NMSS, Non-Motor Symptoms Scale in Parkinson's Disease; p^*^, significant group differences; r, effect sizes of the group differences given by the Pearson correlation coefficient; UPDRS, Unified Parkinson's Disease Rating Scale. Z, Z-score of the paired Wilcoxon signed-rank test.

### 3.2. Data acceptability and reliability

We included patients with PD who completed the NMSS questionnaire at baseline and the 2-year follow-up. The data acceptability of the NMSS at baseline and the 2-year follow-up is shown in Supplementary Tables 1, 2.

Univariate correlation analyses revealed the strongest inter-item correlations were between item 8 (*lack of motivation*) and item 12 (*anhedonia*), with 0.682 at baseline, and between item 10 (*feeling sad*) and item 12 (*anhedonia*), with 0.695 at the 2-year follow-up (Supplementary Tables 3, 4).

The highest Cronbach's alpha coefficients were determined for domain 3 (mood/cognition) at baseline (0.870) and the 2-year follow-up (0.889). Item homogeneity ranged from 0.166 (domain 9, miscellaneous) to 0.534 (domain 3) at baseline and from 0.180 (domain 9) to 0.574 (domain 3) at the 2-year follow-up. The corrected item-total correlations ranged from 0.151 (item 28, *taste/smell*, baseline) to 0.788 (item 10, *feeling sad*, 2-year follow-up). Data on the internal consistency of the NMSS are shown in Supplementary Tables 5, 6.

### 3.3. Network structure

**Figure 1:**
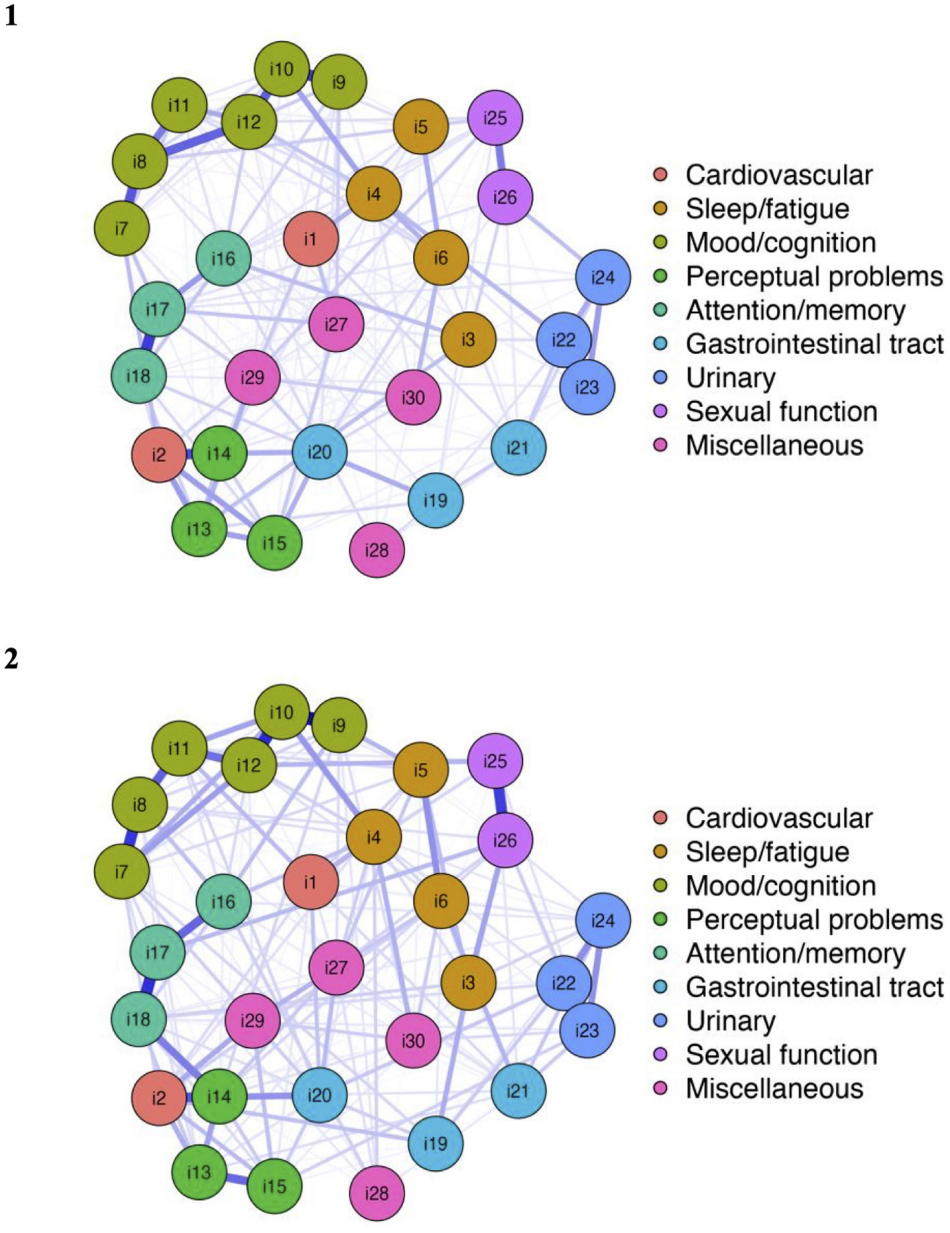
Network structure NMSS items at baseline and the 2-year follow-up.

The respective network plots of the study population at baseline and at the two-year follow-up are shown in Figure 1. The nodes display the items of the NMSS (i1-i30), and the color assignment of the nodes reflects the distribution of the items in the domain structure of the NMSS.

Network analysis revealed well-connected networks at baseline (215 of 435 non-zero edges) and the 2-year follow-up (208 of 435 non-zero edges). The 30 items in the nine domains of the NMSS were not strictly separated. There were various cross-domain associations. In particular, there was a connection between item 2 (*fainting*) and item 14 (*delusions*) and, accordingly, between the cardiovascular domain (domain 1) and the perceptual problems domain (domain 4). On a global level, there were intra-domain associations between the mood/cognition, perceptual problems, attention/memory, urinary, and sexual function domains. However, network analysis revealed that the distribution of items in the cardiovascular and miscellaneous domains did not fully correspond to the visually delimited domain structure. First, it can be noted that there was no strong connection between the item *light headedness* and item *fainting*, which both belong to the cardiovascular domain. Second, items 27–30 of the miscellaneous domain (*pain, taste/smell, weight change*, and *hyperhidrosis*) were not considerably associated with each other. In addition, item 4 (*fatigue*) seems to be associated with both domain 2 (sleep/fatigue) and domain 3 (mood/cognition). Furthermore, the network showed that regarding the gastrointestinal tract domain (domain 5), items 19 and 20 (*sialorrhea* and *dysphagia*) were more connected to each other than to item 21 (*constipation*).

**Figure 2:**
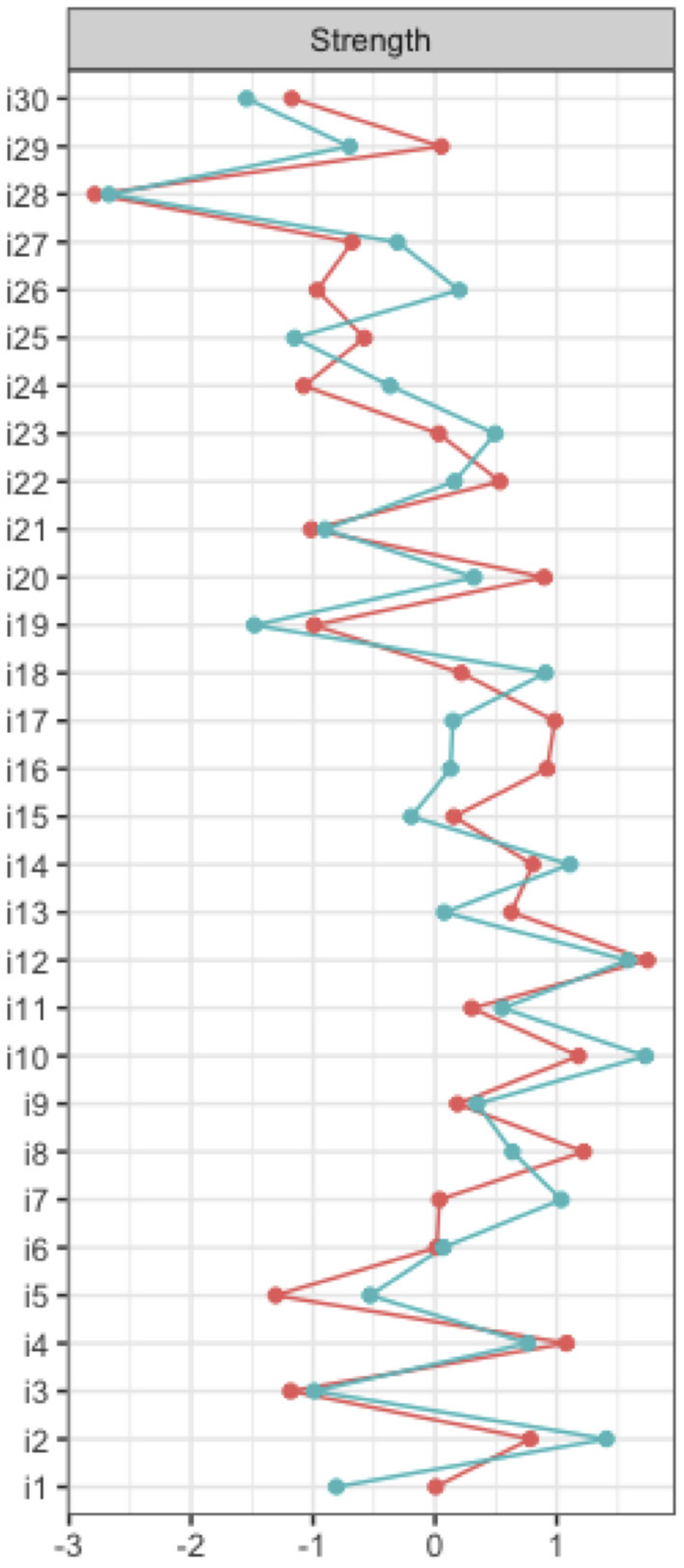
Node strength NMSS items.

For each item of the NMSS, the values of the strength centrality measure at the baseline and 2-year follow-up are shown in Figure 2 (and also tabulated in Supplementary Table 7). At baseline, the highest strength was determined for item 12 (*anhedonia*). This item had the highest input weights from being directly connected other items. At the 2-year follow-up, the strength of item 12 remained high. However, item 10 (*feeling sad*) replaced *anhedonia* as the most central item at the 2-year follow-up. Both items belong to domain 3 of the NMSS (mood/cognition).

Supplemental network analyses at domain level of the NMSS were carried out. Thereby, the highest strength centrality measures were determined for domain 2 (sleep/fatigue) at the baseline and 2-year follow-up (see Supplementary Tables 8, 9). However, these results do not correspond to the findings from the preferred analyses on item level without data reduction.

### 3.4. Network stability

The network accuracy and stability analysis results for the study population at baseline are shown in Supplementary Figures 1–3, and for the study population at the 2-year follow-up in Supplementary Figures 4–6. The case-dropping bootstrapped procedure showed that the centrality measure strength remained sufficiently stable at the baseline [CS(cor = 0.7) = 0.36] and was highly stable at the two-year follow-up [CS(cor = 0.7) = 0.52]. The non-parametric bootstrapped procedure revealed that the identified edge weights are narrow and that, regarding the centrality measure strength, the nodes are significantly different from each other.

### 3.5. Network comparison

As mentioned above, there are some notable differences between the NMSS networks at baseline and the 2-year follow-up. At baseline, the highest strength was determined for item 12 (*anhedonia*). At the 2-year follow-up, item 10 (*feeling sad*) had the highest strength centrality measure. Both items belong to domain 3 of the NMSS (mood/cognition).

In addition, using the network comparison test, network global strength invariance, network structure invariance, and edge strength invariance were compared between the networks at the baseline and the 2-year follow-up based on a permutation test (*n* = 1000). The network comparison test revealed no significant differences in global strength (S = 0.30, *p* = 0.467) or network structure (M = 0.26, *p* = 0.100), as shown in Supplementary Figures 7, 8. Nevertheless, based on the analysis of 435 non-zero edges, a network comparison test revealed 23 individual edge strength differences between the two networks, which are listed in Supplementary Table 10 in more detail. However, these differences are not relevant due to the similarity of global network strength.

## 4. Discussion

The NMSS is frequently used to comprehensively assess a range of non-motor symptoms in patients with PD (5, 6, 27). To the best of our knowledge, this is the first study to examine the NMSS network, characterizing both cross-sectional data and their longitudinal changes.

In preparation for the intended network analysis, we conducted a psychometric evaluation of the NMSS, especially to verify the described limitations of the domain structure within previous validation studies (5, 27). The data acceptability of the NMSS showed a marked floor effect for every item at baseline and at the 2-year follow-up. However, the NMSS was developed as a unified assessment tool for a large variety of non-motor symptoms, including symptoms that may be experienced by only a proportion of patients (5). In accordance with the high prevalence of non-motor symptoms, the total scale was free of a marked floor effect, which was also reported in earlier validation studies (5, 27). In line with the results of the validation study by Martinez-Martin et al. (27), domains 3 (mood/cognition), 5 (attention/memory), and 7 (urinary) reached the highest Cronbach's alpha values. However, our results also confirmed some already known limitations of the domain structure. In our study, the lowest Cronbach's alpha values were obtained for domains 1 (cardiovascular), 6 (gastrointestinal tract), and 9 (miscellaneous). Therefore, item 21 (*constipation*) in domain 6 and item 28 (*taste/smell*) in domain 9 had low corrected item-total correlations and, therefore, low factor loadings. However, limitations of the domain structure were already evident in the original pilot study of the NMSS (5). In particular, the gastrointestinal tract domain showed a weak consistency. Nevertheless, this domain was maintained because it contained relevant symptoms in the digestive area. Accordingly, the importance of the symptoms in real life was considered independent of the statistical results (5).

This study aimed to assess the complex interacting networks of a wide range of non-motor symptoms in PD. For this purpose, we conducted a network analysis based on the 30 items of the NMSS. Our study revealed a well-connected network of non-motor symptoms, indicating that different symptoms were related to each other. Therefore, we were able to show that *anhedonia* (item 12) at baseline and *feeling sad* (item 10) at the 2-year follow-up had the highest strength centrality measures. Although the impact of *anhedonia* remained high during the baseline and 2-year follow-up, the impact of *feeling sad* increased considerably. Both items belong to the mood/cognition domain (domain 3). There was a strong connection between these two items, as determined by the strong inter-item correlations and high edge weights. Accordingly, *anhedonia* and *feeling sad* are considerable associated with several connected non-motor symptoms. This means that positively influencing anhedonia and feelings of sadness may represent a possible therapeutic target to attenuate other non-motor symptoms and, accordingly, improve patients' quality of life.

Again, it should be noted that network analysis is a suitable tool to reveal complex connections between many nodes. Accordingly, one of the main advantages of the analysis is that no data reduction is required. Therefore, the analysis is preferably performed at item level rather than at domain level of the NMSS to retain the greatest possible informative value. However, a large cohort is necessary for this. To further validate these results also in smaller cohorts, it would be important to know whether data reduction from item level to domain level leads to a meaningful loss of information. Our results showed that *anhedonia* and *feeling sad* (which both belong to domain 3, mood/cognition) had the highest strength centrality at the item level, while the sleep/fatigue domain (domain 2) had the highest strength centrality at the domain level. Thus, it should be pointed out that data reduction to the domain level of the NMSS with the intention to perform network analysis also in smaller cohorts should be avoided, especially due to weaknesses of the domain structure of the NMSS.

In general, the number of possible associations within the network increases with the number of variables considered. Accordingly, network analysis of the 30 items of the NMSS revealed numerous associations between the symptoms. However, a central assumption of network analysis is that the overall pattern of connections between nodes (e.g., the 30 non-motor symptoms) is considered to understand the complex interacting system, rather than looking at separate correlations. Nevertheless, the network uncovered individual interesting associations, for example, the association between fainting (item 2) and delusions (item 14). Again, it must be pointed out that this association does not directly correspond to causality as well. As shown in the data acceptability analysis (Supplementary Tables 1, 2), both symptoms had the highest floor effect, and both are known symptoms that appear later in the course of the disease at higher severity (28). Thus, it can be assumed that disease severity mediates this association.

As mentioned above, network analysis revealed high strength centrality measures for *anhedonia* and *feeling sad*. Sad mood is a key symptom of PD-related depression (29). However, data on anhedonia in patients with PD are rare despite anhedonia being a central factor in depression (30). Our study revealed a strong connection between *feeling sad* (item 10) and *anhedonia* (item 12), underlining the impact of depressive symptoms within the network of non-motor symptoms. However, depressive symptoms are heterogeneous and often under-recognized in PD (31, 32). For the successful holistic care of patients with PD, it may be better to consider depression as a spectrum (33), as even subthreshold depression is a frequent problem in patients with PD (34). This is confirmed in the current study, which highlights the influence of depressive symptoms even though BDI-II scores indicated no or low depressiveness for most of the included patients.

A follow-up examination of the patients with PD was conducted after 2 years to examine the progress of motor and non-motor symptoms. In particular, the severity of 23 out of 30 NMSS increased over 2 years; however, the effect sizes were low (Table 1). In addition to the separate longitudinal assessment of each non-motor symptom, we performed a network comparison test. No differences in network global strength and structure were detected, suggesting a stable network over the two-year period.

There are three possible explanations for the high impact of depressive symptoms on the network of non-motor symptoms. First, although the exact pathophysiology of depression in PD is not fully understood, there is evidence of degeneration of the neurotransmitter system, with dopaminergic, noradrenergic, and serotonergic damage contributing to depressive symptoms in PD (35–38). Degeneration of the dopaminergic, noradrenergic, or serotonergic neurotransmitter system can cause various other non-motor symptoms. Second, longitudinal studies have revealed some risk factors that contribute to depression and other non-motor symptoms; thus, many non-motor symptoms share certain risk factors (39, 40), which may explain the interdependence of some symptoms. Third, patients with depression often report somatic symptoms (41, 42). Likewise, it is already known that somatization in PD is associated with a higher NMSS total score (43). Accordingly, there is a higher probability that the somatic symptoms of PD patients with depression may overlap with other non-motor symptoms.

Our study has several limitations. First, the collection of non-motor symptoms was based on a self-report scale, and the perception of symptoms could be biased by mood and motivation. Second, centrality measures of the respective NMSS items should be generalized with caution because they might depend on the studied cohort. The obtained data are not fully representative of the PD population due to the inclusion and exclusion criteria (i.e., age limit, no dementia, no severe comorbidities, and no second-line therapies) (8). Third, estimation of a stable network usually requires a large sample size, which limits its applicability to smaller local cohorts or for further subgroup analyses. Fourth, network analysis remains an exploratory approach, and causal effects cannot be determined.

Taken together, our study revealed that in patients with PD, several non-motor symptoms increased in intensity over time. However, the complex interactions of the 30 NMSS items remained largely stable. *Anhedonia* and *feeling sad* as depressive symptoms had the strongest impact on all non-motor symptoms. Further research is needed to confirm whether influencing anhedonia and feelings of sadness can positively attenuate other non-motor symptoms and improve patients' quality of life.

## Data availability statement

The data analyzed in this study was obtained from the COhort of Patients with Parkinson's DIsease in Spain (COPPADIS) study, the following licenses/restrictions apply: Access to these datasets is subject to approval. Requests to access these datasets should be directed to DS-G, diegosangar@yahoo.es.

## Ethics statement

The study was conducted according to the guidelines of the Declaration of Helsinki, and approved by Comité de Ética de la Investigación Clínica de Galicia from Spain (2014/534; 2/DEC/2014). Written informed consents from all participants in this study were obtained before the start of the study.

## Author contributions

Study concept and design: KH and TP. Data preparation: DS-G. Statistical analysis and interpretation of the data: KH, AS, and TP. First draft of the manuscript: KH. Critical revision of the manuscript: AS, HM, SM, DS-G, and TP. Final approval by all authors.
