# Supplementary material for: Longitudinal analysis of the Non-Motor Symptoms Scale in Parkinson's Disease (NMSS): An exploratory network analysis approach
Source: Front Neurol. 2023 Feb 14;14:972210. doi: 10.3389/fneur.2023.972210 (PMC9971229; doi:10.3389/fneur.2023.972210)
Supplement: Supplementary file 1 [file Data_Sheet_1.PDF]

# *Supplementary Material*

## 1 Supplementary Tables

**Supplement Table 1.:** Data acceptability of the NMSS at baseline.

| | Computable (%) | Median | Min | Max | Floor effect (%) | Ceiling effect (%) |
|---|---|---|---|---|---|---|
| Item 1 | 100 | 0 | 0 | 12 | 59.3 | 0.6 |
| Item 2 | 100 | 0 | 0 | 6 | 93.8 | 0.0 |
| Item 3 | 100 | 1 | 0 | 12 | 45.5 | 2.2 |
| Item 4 | 100 | 1 | 0 | 12 | 39.5 | 3.4 |
| Item 5 | 100 | 0 | 0 | 12 | 64.3 | 3.4 |
| Item 6 | 100 | 0 | 0 | 12 | 59.1 | 1.0 |
| Item 7 | 100 | 0 | 0 | 12 | 74.7 | 0.2 |
| Item 8 | 100 | 0 | 0 | 12 | 65.5 | 1.4 |
| Item 9 | 100 | 0 | 0 | 12 | 53.5 | 3.2 |
| Item 10 | 100 | 0 | 0 | 12 | 53.9 | 1.0 |
| Item 11 | 100 | 0 | 0 | 12 | 67.1 | 1.8 |
| Item 12 | 100 | 0 | 0 | 12 | 62.7 | 1.2 |
| Item 13 | 100 | 0 | 0 | 9 | 86.4 | 0.0 |
| Item 14 | 100 | 0 | 0 | 9 | 90.2 | 0.0 |
| Item 15 | 100 | 0 | 0 | 12 | 87.2 | 1.6 |
| Item 16 | 100 | 0 | 0 | 12 | 52.5 | 1.8 |
| Item 17 | 100 | 0 | 0 | 12 | 56.3 | 0.6 |
| Item 18 | 100 | 0 | 0 | 12 | 68.5 | 0.6 |
| Item 19 | 100 | 0 | 0 | 12 | 58.7 | 0.6 |
| Item 20 | 100 | 0 | 0 | 12 | 77.6 | 0.4 |
| Item 21 | 100 | 0 | 0 | 12 | 59.7 | 2.8 |
| Item 22 | 100 | 1 | 0 | 12 | 40.3 | 5.6 |
| Item 23 | 100 | 1 | 0 | 12 | 45.5 | 3.8 |
| Item 24 | 100 | 1 | 0 | 12 | 37.9 | 3.8 |
| Item 25 | 100 | 0 | 0 | 12 | 61.1 | 8.0 |
| Item 26 | 100 | 0 | 0 | 12 | 58.3 | 8.0 |
| Item 27 | 100 | 0 | 0 | 12 | 70.5 | 2.6 |
| Item 28 | 100 | 2 | 0 | 12 | 41.5 | 11.4 |
| Item 29 | 100 | 0 | 0 | 12 | 80.6 | 1.6 |
| Item 30 | 100 | 0 | 0 | 12 | 66.3 | 4.8 |
| Cardiovascular (domain 1) | 100 | 0 | 0 | 12 | 58.3 | 0.0 |
| Sleep/fatigue (domain 2) | 100 | 6 | 0 | 39 | 15.2 | 0.0 |
| Mood/cognition (domain 3) | 100 | 3 | 0 | 60 | 29.1 | 0.0 |
| Perceptual problems (domain 4) | 100 | 0 | 0 | 27 | 75.2 | 0.0 |
| Attention/memory (domain 5) | 100 | 2 | 0 | 28 | 34.1 | 0.0 |
| Gastrointestinal tract (domain 6) | 100 | 1 | 0 | 36 | 34.7 | 0.2 |
| Urinary (domain 7) | 100 | 6 | 0 | 36 | 17.8 | 1.0 |
| Sexual function (domain 8) | 100 | 1 | 0 | 24 | 43.5 | 3.6 |
| Miscellaneous (domain 9) | 100 | 5 | 0 | 36 | 22.0 | 0.0 |
| NMSS total score | 100 | 34 | 0 | 252 | 0.4 | 0.0 |

Computable data and values of the floor and ceiling effects are given as percentages, and other data are given as absolute values. Assignment of the 30 items to the domain structure of the NMSS: cardiovascular (domain 1; items 1 and 2), sleep/fatigue (domain 2; items 3, 4, 5, and 6), mood/cognition (domain 3; items 7, 8, 9, 10, 11, and 12), perceptual problems (domain 4; items 13, 14, and 15), attention/memory (domain 5; items 16, 17, and 18), gastrointestinal tract (domain 6; items 19, 20, and 21), urinary (domain 7; items 22, 23, and 24), sexual function (domain 8; items 25 and 26), and miscellaneous (domain 9; items 27, 28, 29, and 30). Item 1: light headedness; item 2: fainting; item 3: daytime sleepiness; item 4: fatigue; item 5: sleep initiation; item 6: restless legs; item 7: loss of interest; item 8: lack of motivation; item 9: feeling nervous; item 10: feeling sad; item 11: flat mood; item 12: anhedonia; item 13: hallucinations; item 14: delusions; item 15: diplopia; item 16: concentration; item 17: forgetfulness; item 18: forget to do things; item 19: sialorrhea; item 20: dysphagia; item 21: constipation; item 22: urgency; item 23: frequency; item 24: nocturia; item 25: interest; item 26: problems having sex; item 27: pain; item 28: taste/smell; item 29: weight change; and item 30: hyperhidrosis. NMSS: Non-Motor Symptoms Scale in Parkinson's Disease.

**Supplement Table 2.:** Data acceptability of the NMSS at the two-year follow-up.

| | Computable (%) | Median | Min | Max | Floor effect (%) | Ceiling effect (%) |
|---|---|---|---|---|---|---|
| Item 1 | 100 | 0 | 0 | 12 | 53.5 | 1.0 |
| Item 2 | 100 | 0 | 0 | 6 | 92.8 | 0.2 |
| Item 3 | 100 | 1 | 0 | 12 | 41.5 | 3.2 |
| Item 4 | 100 | 2 | 0 | 12 | 28.7 | 4.2 |
| Item 5 | 100 | 0 | 0 | 12 | 59.3 | 4.2 |
| Item 6 | 100 | 0 | 0 | 12 | 59.5 | 2.2 |
| Item 7 | 100 | 0 | 0 | 12 | 71.3 | 1.4 |
| Item 8 | 100 | 0 | 0 | 12 | 60.5 | 2.6 |
| Item 9 | 100 | 0 | 0 | 12 | 52.5 | 2.0 |
| Item 10 | 100 | 1 | 0 | 12 | 48.9 | 3.0 |
| Item 11 | 100 | 0 | 0 | 12 | 61.3 | 2.0 |
| Item 12 | 100 | 0 | 0 | 12 | 61.1 | 2.2 |
| Item 13 | 100 | 0 | 0 | 12 | 80.4 | 0.8 |
| Item 14 | 100 | 0 | 0 | 12 | 86.0 | 0.4 |
| Item 15 | 100 | 0 | 0 | 12 | 83.0 | 0.6 |
| Item 16 | 100 | 0 | 0 | 12 | 50.7 | 2.0 |
| Item 17 | 100 | 0 | 0 | 12 | 54.1 | 2.0 |
| Item 18 | 100 | 0 | 0 | 12 | 65.1 | 0.8 |
| Item 19 | 100 | 0 | 0 | 12 | 51.7 | 0.6 |
| Item 20 | 100 | 0 | 0 | 12 | 72.9 | 0.4 |
| Item 21 | 100 | 0 | 0 | 12 | 53.3 | 6.6 |
| Item 22 | 100 | 2 | 0 | 12 | 33.5 | 7.6 |
| Item 23 | 100 | 1 | 0 | 12 | 42.1 | 4.4 |
| Item 24 | 100 | 2 | 0 | 12 | 30.1 | 4.4 |
| Item 25 | 100 | 0 | 0 | 12 | 52.9 | 8.8 |
| Item 26 | 100 | 0 | 0 | 12 | 53.7 | 10.2 |
| Item 27 | 100 | 0 | 0 | 12 | 65.1 | 2.2 |
| Item 28 | 100 | 2 | 0 | 12 | 39.1 | 11.4 |
| Item 29 | 100 | 0 | 0 | 12 | 78.4 | 1.0 |
| Item 30 | 100 | 0 | 0 | 12 | 60.9 | 4.2 |
| Cardiovascular (domain 1) | 100 | 0 | 0 | 24 | 52.7 | 0.2 |
| Sleep/fatigue (domain 2) | 100 | 7 | 0 | 44 | 11.0 | 0.0 |
| Mood/cognition (domain 3) | 100 | 4 | 0 | 72 | 25.7 | 0.2 |
| Perceptual problems (domain 4) | 100 | 0 | 0 | 27 | 66.1 | 0.0 |
| Attention/memory (domain 5) | 100 | 2 | 0 | 36 | 30.5 | 0.2 |
| Gastrointestinal tract (domain 6) | 100 | 2 | 0 | 33 | 24.8 | 0.0 |
| Urinary (domain 7) | 100 | 6 | 0 | 36 | 11.6 | 1.8 |
| Sexual function (domain 8) | 100 | 4 | 0 | 24 | 36.9 | 5.6 |
| Miscellaneous (domain 9) | 100 | 6 | 0 | 38 | 19.4 | 0.0 |
| NMSS total score | 100 | 43 | 0 | 229 | 1.4 | 0.0 |

Computable data and values of the floor and ceiling effects are given as percentages, and other data are given as absolute values. Assignment of the 30 items to the domain structure of the NMSS: cardiovascular (domain 1; items 1 and 2), sleep/fatigue (domain 2; items 3, 4, 5, and 6), mood/cognition (domain 3; items 7, 8, 9, 10, 11, and 12), perceptual problems (domain 4; items 13, 14, and 15), attention/memory (domain 5; items 16, 17, and 18), gastrointestinal tract (domain 6; items 19, 20, and 21), urinary (domain 7; items 22, 23, and 24), sexual function (domain 8; items 25 and 26), and miscellaneous (domain 9; items 27, 28, 29, and 30). Item 1: light headedness; item 2: fainting; item 3: daytime sleepiness; item 4: fatigue; item 5: sleep initiation; item 6: restless legs; item 7: loss of interest; item 8: lack of motivation; item 9: feeling nervous; item 10: feeling sad; item 11: flat mood; item 12: anhedonia; item 13: hallucinations; item 14: delusions; item 15: diplopia; item 16: concentration; item 17: forgetfulness; item 18: forget to do things; item 19: sialorrhea; item 20: dysphagia; item 21: constipation; item 22: urgency; item 23: frequency; item 24: nocturia; item 25: interest; item 26: problems having sex; item 27: pain; item 28: taste/smell; item 29: weight change; and item 30: hyperhidrosis. NMSS: Non-Motor Symptoms Scale in Parkinson's Disease.

**Supplement Table 3.:** Inter-item correlation of the NMSS at baseline.

| | 1 | 2 | 3 | 4 | 5 | 6 | 7 | 8 | 9 | 10 | 11 | 12 | 13 | 14 | 15 | 16 | 17 | 18 | 19 | 20 | 21 | 22 | 23 | 24 | 25 | 26 | 27 | 28 | 29 | 30 |
|---|---|---|---|---|---|---|---|---|---|---|---|---|---|---|---|---|---|---|---|---|---|---|---|---|---|---|---|---|---|---|
| Item 1 | 1 | | | | | | | | | | | | | | | | | | | | | | | | | | | | | |
| Item 2 | 0.265 | 1 | | | | | | | | | | | | | | | | | | | | | | | | | | | | |
| Item 3 | 0.215 | 0.163 | 1 | | | | | | | | | | | | | | | | | | | | | | | | | | | |
| Item 4 | 0.394 | 0.137 | 0.249 | 1 | | | | | | | | | | | | | | | | | | | | | | | | | | |
| Item 5 | 0.235 | 0.192 | 0.135 | 0.268 | 1 | | | | | | | | | | | | | | | | | | | | | | | | | |
| Item 6 | 0.258 | 0.220 | 0.137 | 0.406 | 0.311 | 1 | | | | | | | | | | | | | | | | | | | | | | | | |
| Item 7 | 0.147 | 0.337 | 0.146 | 0.275 | 0.149 | 0.18 | 1 | | | | | | | | | | | | | | | | | | | | | | | |
| Item 8 | 0.273 | 0.279 | 0.170 | 0.438 | 0.226 | 0.307 | 0.583 | 1 | | | | | | | | | | | | | | | | | | | | | | |
| Item 9 | 0.335 | 0.178 | 0.230 | 0.445 | 0.263 | 0.314 | 0.394 | 0.446 | 1 | | | | | | | | | | | | | | | | | | | | | |
| Item 10 | 0.370 | 0.206 | 0.168 | 0.525 | 0.292 | 0.365 | 0.450 | 0.582 | 0.628 | 1 | | | | | | | | | | | | | | | | | | | | |
| Item 11 | 0.292 | 0.231 | 0.104 | 0.397 | 0.245 | 0.340 | 0.456 | 0.623 | 0.436 | 0.508 | 1 | | | | | | | | | | | | | | | | | | | |
| Item 12 | 0.360 | 0.281 | 0.186 | 0.528 | 0.252 | 0.400 | 0.480 | 0.682 | 0.513 | 0.657 | 0.568 | 1 | | | | | | | | | | | | | | | | | | |
| Item 13 | 0.299 | 0.530 | 0.309 | 0.206 | 0.102 | 0.260 | 0.278 | 0.302 | 0.261 | 0.267 | 0.324 | 0.332 | 1 | | | | | | | | | | | | | | | | | |
| Item 14 | 0.290 | 0.676 | 0.211 | 0.133 | 0.201 | 0.240 | 0.298 | 0.191 | 0.187 | 0.231 | 0.266 | 0.231 | 0.481 | 1 | | | | | | | | | | | | | | | | |
| Item 15 | 0.248 | 0.395 | 0.178 | 0.293 | 0.186 | 0.168 | 0.194 | 0.175 | 0.130 | 0.151 | 0.253 | 0.184 | 0.476 | 0.349 | 1 | | | | | | | | | | | | | | | |
| Item 16 | 0.275 | 0.205 | 0.310 | 0.374 | 0.233 | 0.307 | 0.293 | 0.422 | 0.391 | 0.411 | 0.365 | 0.428 | 0.329 | 0.266 | 0.34 | 1 | | | | | | | | | | | | | | |
| Item 17 | 0.222 | 0.301 | 0.242 | 0.228 | 0.173 | 0.282 | 0.349 | 0.288 | 0.246 | 0.220 | 0.223 | 0.237 | 0.268 | 0.325 | 0.278 | 0.438 | 1 | | | | | | | | | | | | | |
| Item 18 | 0.152 | 0.367 | 0.242 | 0.182 | 0.089 | 0.226 | 0.327 | 0.273 | 0.192 | 0.208 | 0.160 | 0.293 | 0.386 | 0.350 | 0.277 | 0.390 | 0.560 | 1 | | | | | | | | | | | | |
| Item 19 | 0.209 | 0.244 | 0.156 | 0.107 | 0.057 | 0.139 | 0.209 | 0.209 | 0.177 | 0.203 | 0.138 | 0.188 | 0.288 | 0.218 | 0.162 | 0.271 | 0.206 | 0.283 | 1 | | | | | | | | | | | |
| Item 20 | 0.326 | 0.408 | 0.230 | 0.305 | 0.166 | 0.302 | 0.351 | 0.289 | 0.286 | 0.345 | 0.293 | 0.330 | 0.369 | 0.405 | 0.321 | 0.271 | 0.270 | 0.256 | 0.350 | 1 | | | | | | | | | | |
| Item 21 | 0.294 | 0.146 | 0.119 | 0.201 | 0.148 | 0.236 | 0.090 | 0.085 | 0.129 | 0.111 | 0.168 | 0.163 | 0.117 | 0.202 | 0.159 | 0.125 | 0.150 | 0.154 | 0.196 | 0.284 | 1 | | | | | | | | | |
| Item 22 | 0.197 | 0.076 | 0.209 | 0.327 | 0.064 | 0.136 | 0.135 | 0.221 | 0.182 | 0.179 | 0.187 | 0.240 | 0.129 | 0.100 | 0.167 | 0.183 | 0.181 | 0.202 | 0.185 | 0.154 | 0.228 | 1 | | | | | | | | |
| Item 23 | 0.199 | 0.098 | 0.149 | 0.288 | 0.108 | 0.188 | 0.141 | 0.208 | 0.198 | 0.197 | 0.213 | 0.238 | 0.136 | 0.114 | 0.188 | 0.154 | 0.203 | 0.260 | 0.142 | 0.203 | 0.192 | 0.649 | 1 | | | | | | | |
| Item 24 | 0.139 | 0.100 | 0.211 | 0.201 | 0.045 | 0.176 | 0.101 | 0.197 | 0.097 | 0.123 | 0.139 | 0.228 | 0.179 | 0.090 | 0.145 | 0.131 | 0.194 | 0.199 | 0.107 | 0.130 | 0.220 | 0.411 | 0.379 | 1 | | | | | | |
| Item 25 | 0.189 | 0.090 | 0.126 | 0.259 | 0.188 | 0.196 | 0.239 | 0.320 | 0.227 | 0.259 | 0.240 | 0.302 | 0.209 | 0.112 | 0.100 | 0.247 | 0.240 | 0.147 | 0.219 | 0.205 | 0.168 | 0.160 | 0.099 | 0.166 | 1 | | | | | |
| Item 26 | 0.154 | 0.097 | 0.200 | 0.213 | 0.098 | 0.129 | 0.103 | 0.192 | 0.051 | 0.102 | 0.176 | 0.177 | 0.109 | 0.120 | 0.082 | 0.172 | 0.204 | 0.122 | 0.121 | 0.190 | 0.201 | 0.204 | 0.164 | 0.267 | 0.385 | 1 | | | | |
| Item 27 | 0.272 | 0.197 | 0.195 | 0.250 | 0.131 | 0.239 | 0.244 | 0.251 | 0.309 | 0.273 | 0.195 | 0.273 | 0.256 | 0.203 | 0.289 | 0.293 | 0.285 | 0.219 | 0.164 | 0.167 | 0.151 | 0.124 | 0.140 | 0.056 | 0.128 | 0.110 | 1 | | | |
| Item 28 | 0.079 | 0.007 | 0.091 | 0.157 | 0.056 | 0.143 | 0.108 | 0.136 | 0.087 | 0.122 | 0.097 | 0.147 | 0.068 | 0.010 | 0.065 | 0.189 | 0.129 | 0.049 | 0.143 | 0.081 | 0.151 | 0.131 | 0.140 | 0.107 | 0.135 | 0.072 | 0.084 | 1 | | |
| Item 29 | 0.363 | 0.282 | 0.143 | 0.289 | 0.172 | 0.221 | 0.238 | 0.278 | 0.307 | 0.336 | 0.274 | 0.210 | 0.367 | 0.287 | 0.237 | 0.215 | 0.325 | 0.280 | 0.187 | 0.315 | 0.181 | 0.121 | 0.127 | 0.091 | 0.247 | 0.094 | 0.275 | 0.070 | 1 | |
| Item 30 | 0.166 | 0.145 | 0.046 | 0.290 | 0.183 | 0.293 | 0.104 | 0.187 | 0.185 | 0.255 | 0.241 | 0.200 | 0.206 | 0.166 | 0.204 | 0.221 | 0.143 | 0.138 | 0.144 | 0.241 | 0.066 | 0.162 | 0.207 | 0.090 | 0.127 | 0.148 | 0.189 | 0.148 | 0.228 | 1 |

Item 1: light headedness; item 2: fainting; item 3: daytime sleepiness; item 4: fatigue; item 5: sleep initiation; item 6: restless legs; item 7: loss of interest; item 8: lack of motivation; item 9: feeling nervous; item 10: feeling sad; item 11: flat mood; item 12: anhedonia; item 13: hallucinations; item 14: delusions; item 15: diplopia; item 16: concentration; item 17: forgetfulness; item 18: forget to do things; item 19: sialorrhea; item 20: dysphagia; item 21: constipation; item 22: urgency; item 23: frequency; item 24: nocturia; item 25: interest; item 26: problems having sex; item 27: pain; item 28: taste/smell; item 29: weight change; and item 30: hyperhidrosis. NMSS: Non-Motor Symptoms Scale in Parkinson's Disease.

**Supplement Table 4.:** Inter-item correlation of the NMSS at the two-year follow-up.

| | 1 | 2 | 3 | 4 | 5 | 6 | 7 | 8 | 9 | 10 | 11 | 12 | 13 | 14 | 15 | 16 | 17 | 18 | 19 | 20 | 21 | 22 | 23 | 24 | 25 | 26 | 27 | 28 | 29 | 30 |
|---|---|---|---|---|---|---|---|---|---|---|---|---|---|---|---|---|---|---|---|---|---|---|---|---|---|---|---|---|---|---|
| Item 1 | 1 | | | | | | | | | | | | | | | | | | | | | | | | | | | | | |
| Item 2 | 0.351 | 1 | | | | | | | | | | | | | | | | | | | | | | | | | | | | |
| Item 3 | 0.162 | 0.155 | 1 | | | | | | | | | | | | | | | | | | | | | | | | | | | |
| Item 4 | 0.340 | 0.176 | 0.272 | 1 | | | | | | | | | | | | | | | | | | | | | | | | | | |
| Item 5 | 0.249 | 0.239 | 0.282 | 0.251 | 1 | | | | | | | | | | | | | | | | | | | | | | | | | |
| Item 6 | 0.236 | 0.343 | 0.210 | 0.331 | 0.367 | 1 | | | | | | | | | | | | | | | | | | | | | | | | |
| Item 7 | 0.183 | 0.383 | 0.130 | 0.405 | 0.333 | 0.312 | 1 | | | | | | | | | | | | | | | | | | | | | | | |
| Item 8 | 0.224 | 0.315 | 0.097 | 0.399 | 0.237 | 0.235 | 0.665 | 1 | | | | | | | | | | | | | | | | | | | | | | |
| Item 9 | 0.215 | 0.293 | 0.148 | 0.418 | 0.365 | 0.263 | 0.505 | 0.444 | 1 | | | | | | | | | | | | | | | | | | | | | |
| Item 10 | 0.345 | 0.270 | 0.103 | 0.498 | 0.362 | 0.272 | 0.629 | 0.553 | 0.668 | 1 | | | | | | | | | | | | | | | | | | | | |
| Item 11 | 0.340 | 0.319 | 0.116 | 0.352 | 0.291 | 0.227 | 0.508 | 0.584 | 0.487 | 0.598 | 1 | | | | | | | | | | | | | | | | | | | |
| Item 12 | 0.339 | 0.326 | 0.114 | 0.474 | 0.280 | 0.302 | 0.571 | 0.527 | 0.574 | 0.695 | 0.599 | 1 | | | | | | | | | | | | | | | | | | |
| Item 13 | 0.271 | 0.514 | 0.181 | 0.155 | 0.190 | 0.249 | 0.343 | 0.312 | 0.236 | 0.292 | 0.276 | 0.303 | 1 | | | | | | | | | | | | | | | | | |
| Item 14 | 0.192 | 0.541 | 0.235 | 0.262 | 0.238 | 0.262 | 0.355 | 0.331 | 0.317 | 0.283 | 0.305 | 0.342 | 0.536 | 1 | | | | | | | | | | | | | | | | |
| Item 15 | 0.276 | 0.529 | 0.192 | 0.204 | 0.157 | 0.287 | 0.244 | 0.218 | 0.183 | 0.204 | 0.243 | 0.228 | 0.481 | 0.428 | 1 | | | | | | | | | | | | | | | |
| Item 16 | 0.221 | 0.205 | 0.194 | 0.361 | 0.218 | 0.192 | 0.361 | 0.410 | 0.374 | 0.406 | 0.325 | 0.416 | 0.277 | 0.335 | 0.182 | 1 | | | | | | | | | | | | | | |
| Item 17 | 0.122 | 0.221 | 0.202 | 0.226 | 0.214 | 0.177 | 0.340 | 0.349 | 0.288 | 0.279 | 0.214 | 0.313 | 0.293 | 0.412 | 0.136 | 0.501 | 1 | | | | | | | | | | | | | |
| Item 18 | 0.184 | 0.318 | 0.247 | 0.246 | 0.114 | 0.216 | 0.264 | 0.290 | 0.242 | 0.197 | 0.243 | 0.243 | 0.419 | 0.568 | 0.329 | 0.415 | 0.603 | 1 | | | | | | | | | | | | |
| Item 19 | 0.179 | 0.298 | 0.249 | 0.205 | 0.109 | 0.229 | 0.252 | 0.177 | 0.154 | 0.221 | 0.164 | 0.216 | 0.280 | 0.228 | 0.278 | 0.231 | 0.158 | 0.229 | 1 | | | | | | | | | | | |
| Item 20 | 0.280 | 0.487 | 0.147 | 0.306 | 0.211 | 0.255 | 0.358 | 0.274 | 0.312 | 0.294 | 0.355 | 0.317 | 0.344 | 0.393 | 0.403 | 0.273 | 0.288 | 0.325 | 0.374 | 1 | | | | | | | | | | |
| Item 21 | 0.198 | 0.200 | 0.176 | 0.190 | 0.232 | 0.289 | 0.241 | 0.274 | 0.224 | 0.226 | 0.241 | 0.185 | 0.252 | 0.208 | 0.154 | 0.232 | 0.245 | 0.223 | 0.148 | 0.153 | 1 | | | | | | | | | |
| Item 22 | 0.201 | 0.107 | 0.237 | 0.275 | 0.095 | 0.183 | 0.107 | 0.179 | 0.159 | 0.205 | 0.160 | 0.202 | 0.138 | 0.187 | 0.134 | 0.231 | 0.241 | 0.292 | 0.240 | 0.280 | 0.199 | 1 | | | | | | | | |
| Item 23 | 0.177 | 0.169 | 0.209 | 0.236 | 0.073 | 0.210 | 0.166 | 0.165 | 0.209 | 0.169 | 0.173 | 0.211 | 0.179 | 0.232 | 0.262 | 0.220 | 0.223 | 0.317 | 0.193 | 0.261 | 0.237 | 0.573 | 1 | | | | | | | |
| Item 24 | 0.189 | 0.212 | 0.247 | 0.257 | 0.180 | 0.248 | 0.170 | 0.251 | 0.221 | 0.175 | 0.191 | 0.173 | 0.205 | 0.180 | 0.175 | 0.267 | 0.201 | 0.265 | 0.202 | 0.226 | 0.266 | 0.447 | 0.457 | 1 | | | | | | |
| Item 25 | 0.187 | 0.119 | 0.122 | 0.237 | 0.103 | 0.144 | 0.161 | 0.158 | 0.148 | 0.225 | 0.251 | 0.295 | 0.081 | 0.157 | 0.189 | 0.165 | 0.126 | 0.183 | 0.150 | 0.168 | 0.206 | 0.220 | 0.247 | 0.188 | 1 | | | | | |
| Item 26 | 0.101 | 0.086 | 0.272 | 0.191 | 0.126 | 0.165 | 0.161 | 0.221 | 0.077 | 0.119 | 0.202 | 0.219 | 0.183 | 0.268 | 0.155 | 0.207 | 0.231 | 0.238 | 0.205 | 0.157 | 0.210 | 0.272 | 0.284 | 0.277 | 0.484 | 1 | | | | |
| Item 27 | 0.232 | 0.293 | 0.161 | 0.313 | 0.265 | 0.286 | 0.254 | 0.207 | 0.333 | 0.231 | 0.309 | 0.262 | 0.191 | 0.305 | 0.273 | 0.148 | 0.180 | 0.213 | 0.167 | 0.365 | 0.258 | 0.128 | 0.148 | 0.179 | 0.149 | 0.202 | 1 | | | |
| Item 28 | 0.087 | 0.075 | 0.066 | 0.214 | 0.083 | 0.166 | 0.179 | 0.195 | 0.200 | 0.181 | 0.143 | 0.177 | 0.088 | 0.155 | 0.099 | 0.237 | 0.175 | 0.141 | 0.071 | 0.118 | 0.170 | 0.121 | 0.101 | 0.044 | 0.086 | 0.081 | 0.166 | 1 | | |
| Item 29 | 0.222 | 0.386 | 0.103 | 0.22 | 0.241 | 0.294 | 0.314 | 0.152 | 0.209 | 0.217 | 0.243 | 0.289 | 0.273 | 0.298 | 0.307 | 0.200 | 0.233 | 0.261 | 0.218 | 0.370 | 0.126 | 0.107 | 0.207 | 0.116 | 0.181 | 0.195 | 0.274 | 0.108 | 1 | |
| Item 30 | 0.124 | 0.191 | 0.097 | 0.288 | 0.173 | 0.261 | 0.231 | 0.137 | 0.231 | 0.176 | 0.169 | 0.177 | 0.171 | 0.250 | 0.179 | 0.214 | 0.160 | 0.213 | 0.117 | 0.232 | 0.204 | 0.138 | 0.247 | 0.117 | 0.179 | 0.115 | 0.166 | 0.127 | 0.239 | 1 |

Item 1: light headedness; item 2: fainting; item 3: daytime sleepiness; item 4: fatigue; item 5: sleep initiation; item 6: restless legs; item 7: loss of interest; item 8: lack of motivation; item 9: feeling nervous; item 10: feeling sad; item 11: flat mood; item 12: anhedonia; item 13: hallucinations; item 14: delusions; item 15: diplopia; item 16: concentration; item 17: forgetfulness; item 18: forget to do things; item 19: sialorrhea; item 20: dysphagia; item 21: constipation; item 22: urgency; item 23: frequency; item 24: nocturia; item 25: interest; item 26: problems having sex; item 27: pain; item 28: taste/smell; item 29: weight change; and item 30: hyperhidrosis. NMSS: Non-Motor Symptoms Scale in Parkinson's Disease.

**Supplement Table 5.:** Internal consistency of the NMSS at baseline.

| | Cronbach's alpha | Item homogeneity | Corrected item-total correlation | Cronbach's alpha if item deleted |
|---|---|---|---|---|
| Cardiovascular (domain 1) | 0.322 | 0.265 | | |
| Item 1 | | | 0.265 | / |
| Item 2 | | | 0.265 | / |
| Sleep/fatigue (domain 2) | 0.573 | 0.251 | | |
| Item 3 | | | 0.238 | 0.588 |
| Item 4 | | | 0.447 | 0.418 |
| Item 5 | | | 0.331 | 0.521 |
| Item 6 | | | 0.419 | 0.457 |
| Mood/cognition (domain 3) | 0.870 | 0.534 | | |
| Item 7 | | | 0.583 | 0.864 |
| Item 8 | | | 0.739 | 0.836 |
| Item 9 | | | 0.605 | 0.863 |
| Item 10 | | | 0.729 | 0.838 |
| Item 11 | | | 0.649 | 0.852 |
| Item 12 | | | 0.746 | 0.835 |
| Perceptual problems (domain 4) | 0.672 | 0.435 | | |
| Item 13 | | | 0.574 | 0.470 |
| Item 14 | | | 0.469 | 0.625 |
| Item 15 | | | 0.486 | 0.641 |
| Attention/memory (domain 5) | 0.704 | 0.463 | | |
| Item 16 | | | 0.470 | 0.716 |
| Item 17 | | | 0.583 | 0.540 |
| Item 18 | | | 0.548 | 0.599 |
| Gastrointestinal tract (domain 6) | 0.485 | 0.276 | | |
| Item 19 | | | 0.305 | 0.385 |
| Item 20 | | | 0.400 | 0.316 |
| Item 21 | | | 0.282 | 0.501 |
| Urinary (domain 7) | 0.736 | 0.480 | | |
| Item 22 | | | 0.639 | 0.550 |
| Item 23 | | | 0.620 | 0.581 |
| Item 24 | | | 0.436 | 0.785 |
| Sexual function (domain 8) | 0.556 | 0.385 | | |
| Item 25 | | | 0.385 | / |
| Item 26 | | | 0.385 | / |
| Miscellaneous (domain 9) | 0.398 | 0.166 | | |
| Item 27 | | | 0.243 | 0.309 |
| Item 28 | | | 0.151 | 0.455 |
| Item 29 | | | 0.268 | 0.311 |
| Item 30 | | | 0.272 | 0.271 |

Item 1: light headedness; item 2: fainting; item 3: daytime sleepiness; item 4: fatigue; item 5: sleep initiation; item 6: restless legs; item 7: loss of interest; item 8: lack of motivation; item 9: feeling nervous; item 10: feeling sad; item 11: flat mood; item 12: anhedonia; item 13: hallucinations; item 14: delusions; item 15: diplopia; item 16: concentration; item 17: forgetfulness; item 18: forget to do things; item 19: sialorrhea; item 20: dysphagia; item 21: constipation; item 22: urgency; item 23: frequency; item 24: nocturia; item 25: interest; item 26: problems having sex; item 27: pain; item 28: taste/smell; item 29: weight change; and item 30: hyperhidrosis. NMSS: Non-Motor Symptoms Scale in Parkinson's Disease.

**Supplement Table 6.:** Internal consistency of the NMSS at the two-year follow-up.

| | Cronbach's alpha | Item homogeneity | Corrected item-total correlation | Cronbach's alpha if item deleted |
|---|---|---|---|---|
| Cardiovascular (domain 1) | 0.424 | 0.351 | | |
| Item 1 | | | 0.351 | / |
| Item 2 | | | 0.351 | / |
| Sleep/fatigue (domain 2) | 0.611 | 0.285 | | |
| Item 3 | | | 0.349 | 0.571 |
| Item 4 | | | 0.389 | 0.545 |
| Item 5 | | | 0.411 | 0.525 |
| Item 6 | | | 0.425 | 0.522 |
| Mood/cognition (domain 3) | 0.889 | 0.574 | | |
| Item 7 | | | 0.711 | 0.870 |
| Item 8 | | | 0.672 | 0.875 |
| Item 9 | | | 0.655 | 0.878 |
| Item 10 | | | 0.788 | 0.855 |
| Item 11 | | | 0.681 | 0.873 |
| Item 12 | | | 0.736 | 0.864 |
| Perceptual problems (domain 4) | 0.735 | 0.482 | | |
| Item 13 | | | 0.600 | 0.598 |
| Item 14 | | | 0.560 | 0.649 |
| Item 15 | | | 0.519 | 0.697 |
| Attention/memory (domain 5) | 0.747 | 0.506 | | |
| Item 16 | | | 0.515 | 0.747 |
| Item 17 | | | 0.647 | 0.575 |
| Item 18 | | | 0.582 | 0.666 |
| Gastrointestinal tract (domain 6) | 0.384 | 0.225 | | |
| Item 19 | | | 0.282 | 0.218 |
| Item 20 | | | 0.305 | 0.231 |
| Item 21 | | | 0.181 | 0.537 |
| Urinary (domain 7) | 0.743 | 0.493 | | |
| Item 22 | | | 0.598 | 0.628 |
| Item 23 | | | 0.610 | 0.615 |
| Item 24 | | | 0.509 | 0.725 |
| Sexual function (domain 8) | 0.651 | 0.484 | | |
| Item 25 | | | 0.484 | / |
| Item 26 | | | 0.484 | / |
| Miscellaneous (domain 9) | 0.422 | 0.180 | | |
| Item 27 | | | 0.281 | 0.312 |
| Item 28 | | | 0.195 | 0.438 |
| Item 29 | | | 0.294 | 0.337 |
| Item 30 | | | 0.239 | 0.348 |

Item 1: light headedness; item 2: fainting; item 3: daytime sleepiness; item 4: fatigue; item 5: sleep initiation; item 6: restless legs; item 7: loss of interest; item 8: lack of motivation; item 9: feeling nervous; item 10: feeling sad; item 11: flat mood; item 12: anhedonia; item 13: hallucinations; item 14: delusions; item 15: diplopia; item 16: concentration; item 17: forgetfulness; item 18: forget to do things; item 19: sialorrhea; item 20: dysphagia; item 21: constipation; item 22: urgency; item 23: frequency; item 24: nocturia; item 25: interest; item 26: problems having sex; item 27: pain; item 28: taste/smell; item 29: weight change; and item 30: hyperhidrosis. NMSS: Non-Motor Symptoms Scale in Parkinson's Disease.

**Supplement Table 7.:** Node strength NMSS items.

| Node | Strength baseline | Strength two-year follow-up |
|---|---|---|
| i1 | 0.006 | -0.809 |
| i2 | 0.782 | 1.407 |
| i3 | -1.184 | -0.990 |
| i4 | 1.079 | 0.762 |
| i5 | -1.306 | -0.532 |
| i6 | 0.014 | 0.069 |
| i7 | 0.037 | 1.036 |
| i8 | 1.220 | 0.634 |
| i9 | 0.184 | 0.346 |
| i10 | 1.178 | 1.728 |
| i11 | 0.301 | 0.552 |
| i12 | 1.744 | 1.582 |
| i13 | 0.625 | 0.076 |
| i14 | 0.803 | 1.108 |
| i15 | 0.154 | -0.195 |
| i16 | 0.920 | 0.128 |
| i17 | 0.983 | 0.147 |
| i18 | 0.217 | 0.906 |
| i19 | -0.991 | -1.481 |
| i20 | 0.896 | 0.321 |
| i21 | -1.016 | -0.902 |
| i22 | 0.532 | 0.161 |
| i23 | 0.032 | 0.494 |
| i24 | -1.076 | -0.369 |
| i25 | -0.578 | -1.152 |
| i26 | -0.966 | 0.200 |
| i27 | -0.680 | -0.307 |
| i28 | -2.790 | -2.673 |
| i29 | 0.053 | -0.700 |
| i30 | -1.174 | -1.546 |

The nodes display the NMSS items (i1-i30). Item 1: light headedness; item 2: fainting; item 3: daytime sleepiness; item 4: fatigue; item 5: sleep initiation; item 6: restless legs; item 7: loss of interest; item 8: lack of motivation; item 9: feeling nervous; item 10: feeling sad; item 11: flat mood; item 12: anhedonia; item 13: hallucinations; item 14: delusions; item 15: diplopia; item 16: concentration; item 17: forgetfulness; item 18: forget to do things; item 19: sialorrhea; item 20: dysphagia; item 21: constipation; item 22: urgency; item 23: frequency; item 24: nocturia; item 25: interest; item 26: problems having sex; item 27: pain; item 28: taste/smell; item 29: weight change; and item 30: hyperhidrosis.

**Supplement Table 8.:** Node strength NMSS domains.

| Node | Strength baseline | Strength two-year follow-up |
|---|---|---|
| D1 | -0.564 | -1.161 |
| D2 | 1.698 | 1.438 |
| D3 | 1.140 | 1.002 |
| D4 | -0.289 | -0.309 |
| D5 | 0.790 | 0.621 |
| D6 | -0.082 | 0.747 |
| D7 | -1.164 | -0.259 |
| D8 | -1.123 | -1.436 |
| D9 | -0.407 | -0.643 |

The nodes display the NMSS domains (D1-D9). Domain 1: cardiovascular (items 1 and 2); Domain 2: sleep/fatigue (items 3, 4, 5, and 6); Domain 3: mood/cognition (items 7, 8, 9, 10, 11, and 12); Domain 4: perceptual problems (items 13, 14, and 15); Domain 5: attention/memory (items 16, 17, and 18); Domain 6: gastrointestinal tract (items 19, 20, and 21), Domain 7: urinary (items 22, 23, and 24); Domain 8: sexual function (items 25 and 26); Domain 9: miscellaneous (items 27, 28, 29, and 30). Item 1: light headedness; item 2: fainting; item 3: daytime sleepiness; item 4: fatigue; item 5: sleep initiation; item 6: restless legs; item 7: loss of interest; item 8: lack of motivation; item 9: feeling nervous; item 10: feeling sad; item 11: flat mood; item 12: anhedonia; item 13: hallucinations; item 14: delusions; item 15: diplopia; item 16: concentration; item 17: forgetfulness; item 18: forget to do things; item 19: sialorrhea; item 20: dysphagia; item 21: constipation; item 22: urgency; item 23: frequency; item 24: nocturia; item 25: interest; item 26: problems having sex; item 27: pain; item 28: taste/smell; item 29: weight change; and item 30: hyperhidrosis.

**Supplement Table 9.:** Edge weights NMSS domains.

| Baseline | | | | | | | | | |
|---|---|---|---|---|---|---|---|---|---|
| Node | D1 | D2 | D3 | D4 | D5 | D6 | D7 | D8 | D9 |
| D1 | 0.000 | 0.157 | 0.121 | 0.196 | 0.050 | 0.060 | 0.018 | 0.013 | 0.035 |
| D2 | 0.157 | 0.000 | 0.326 | 0.129 | 0.104 | 0.061 | 0.111 | 0.090 | 0.108 |
| D3 | 0.121 | 0.326 | 0.000 | 0.040 | 0.217 | 0.031 | 0.049 | 0.068 | 0.127 |
| D4 | 0.196 | 0.129 | 0.040 | 0.000 | 0.135 | 0.077 | 0.040 | 0.000 | 0.087 |
| D5 | 0.050 | 0.104 | 0.217 | 0.135 | 0.000 | 0.117 | 0.050 | 0.111 | 0.128 |
| D6 | 0.060 | 0.061 | 0.031 | 0.077 | 0.117 | 0.000 | 0.174 | 0.116 | 0.108 |
| D7 | 0.018 | 0.111 | 0.049 | 0.040 | 0.050 | 0.174 | 0.000 | 0.075 | 0.018 |
| D8 | 0.013 | 0.090 | 0.068 | 0.000 | 0.111 | 0.116 | 0.075 | 0.000 | 0.069 |
| D9 | 0.035 | 0.108 | 0.127 | 0.087 | 0.128 | 0.108 | 0.018 | 0.069 | 0.000 |
| Two-year follow-up | | | | | | | | | |
| Node | D1 | D2 | D3 | D4 | D5 | D6 | D7 | D8 | D9 |
| D1 | 0.000 | 0.150 | 0.092 | 0.157 | 0.000 | 0.089 | 0.021 | 0.032 | 0.052 |
| D2 | 0.150 | 0.000 | 0.290 | 0.027 | 0.046 | 0.120 | 0.131 | 0.070 | 0.188 |
| D3 | 0.092 | 0.290 | 0.000 | 0.101 | 0.271 | 0.049 | 0.000 | 0.063 | 0.085 |
| D4 | 0.157 | 0.027 | 0.101 | 0.000 | 0.153 | 0.121 | 0.068 | 0.008 | 0.099 |
| D5 | 0.000 | 0.046 | 0.271 | 0.153 | 0.000 | 0.082 | 0.120 | 0.106 | 0.109 |
| D6 | 0.089 | 0.120 | 0.049 | 0.121 | 0.082 | 0.000 | 0.243 | 0.084 | 0.121 |
| D7 | 0.021 | 0.131 | 0.000 | 0.068 | 0.120 | 0.243 | 0.000 | 0.159 | 0.000 |
| D8 | 0.032 | 0.070 | 0.063 | 0.008 | 0.106 | 0.084 | 0.159 | 0.000 | 0.026 |
| D9 | 0.052 | 0.188 | 0.085 | 0.099 | 0.109 | 0.121 | 0.000 | 0.026 | 0.000 |

The nodes display the NMSS domains (D1-D9). Domain 1: cardiovascular (items 1 and 2); Domain 2: sleep/fatigue (items 3, 4, 5, and 6); Domain 3: mood/cognition (items 7, 8, 9, 10, 11, and 12); Domain 4: perceptual problems (items 13, 14, and 15); Domain 5: attention/memory (items 16, 17, and 18); Domain 6: gastrointestinal tract (items 19, 20, and 21), Domain 7: urinary (items 22, 23, and 24); Domain 8: sexual function (items 25 and 26); Domain 9: miscellaneous (items 27, 28, 29, and 30). Item 1: light headedness; item 2: fainting; item 3: daytime sleepiness; item 4: fatigue; item 5: sleep initiation; item 6: restless legs; item 7: loss of interest; item 8: lack of motivation; item 9: feeling nervous; item 10: feeling sad; item 11: flat mood; item 12: anhedonia; item 13: hallucinations; item 14: delusions; item 15: diplopia; item 16: concentration; item 17: forgetfulness; item 18: forget to do things; item 19: sialorrhea; item 20: dysphagia; item 21: constipation; item 22: urgency; item 23: frequency; item 24: nocturia; item 25: interest; item 26: problems having sex; item 27: pain; item 28: taste/smell; item 29: weight change; and item 30: hyperhidrosis.

**Supplement Table 10.:** Significant individual edge weight differences in the network comparison test.

| Node 1 | Node 2 | p |
|---|---|---|
| i2 | i18 | 0.002 |
| i8 | i12 | 0.003 |
| i8 | i25 | 0.003 |
| i3 | i13 | 0.004 |
| i20 | i21 | 0.004 |
| i16 | i27 | 0.014 |
| i14 | i18 | 0.017 |
| i20 | i27 | 0.019 |
| i12 | i24 | 0.020 |
| i23 | i25 | 0.020 |
| i1 | i14 | 0.021 |
| i15 | i16 | 0.022 |
| i15 | i17 | 0.025 |
| i17 | i25 | 0.025 |
| i10 | i20 | 0.027 |
| i16 | i24 | 0.028 |
| i8 | i21 | 0.031 |
| i12 | i29 | 0.031 |
| i17 | i27 | 0.032 |
| i2 | i14 | 0.038 |
| i13 | i25 | 0.038 |
| i22 | i23 | 0.046 |
| i3 | i16 | 0.049 |

The nodes display the NMSS items (i1-i30). Item 1: light headedness; item 2: fainting; item 3: daytime sleepiness; item 4: fatigue; item 5: sleep initiation; item 6: restless legs; item 7: loss of interest; item 8: lack of motivation; item 9: feeling nervous; item 10: feeling sad; item 11: flat mood; item 12: anhedonia; item 13: hallucinations; item 14: delusions; item 15: diplopia; item 16: concentration; item 17: forgetfulness; item 18: forget to do things; item 19: sialorrhea; item 20: dysphagia; item 21: constipation; item 22: urgency; item 23: frequency; item 24: nocturia; item 25: interest; item 26: problems having sex; item 27: pain; item 28: taste/smell; item 29: weight change; and item 30: hyperhidrosis.

## 2 Supplementary Figures

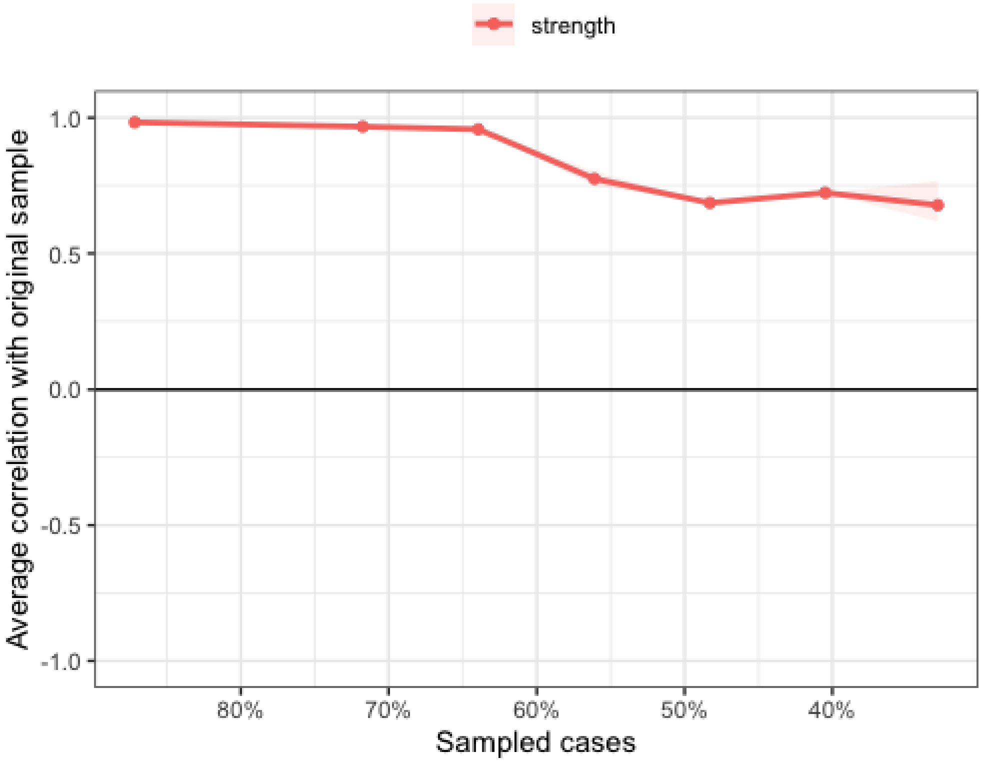


**Supplement Figure 1.:** Case-dropping bootstrapped procedure of strength at baseline (number of bootstraps = 1000). The correlations of the centrality measure strength between the original sample and those from the subsamples with an increasingly higher percentage of dropout cases were calculated. The correlation stability coefficient (CS coefficient) represents the maximum proportion of cases that can be dropped to retain a correlation of at least 0.70 with the original strength in at least 95% of the samples. The 95% confidence interval of the correlation is indicated. The case-dropping bootstrap procedure showed that CS coefficient of node strength (CS(cor=0.7) = 0.36) remained sufficient.

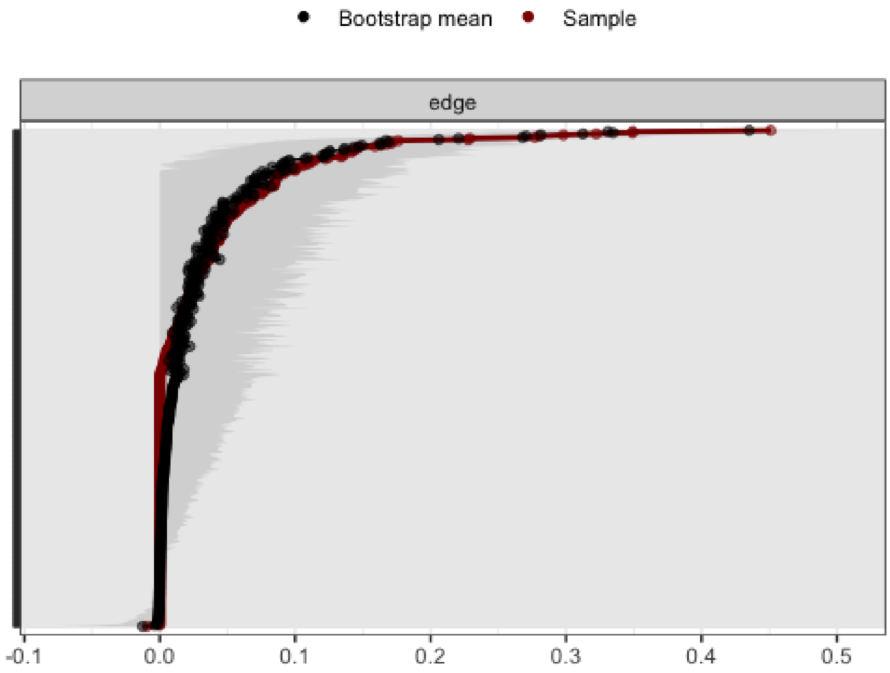


**Supplement Figure 2.:** Nonparametric bootstrapped procedure of edge weights at baseline (number of bootstraps = 1000). Using the nonparametric bootstrapping procedure, the 95% bootstrapped confidence interval of the edge weights was determined around the regularized edge weights. Each horizontal line represents an edge. Y-axis labeling was omitted to avoid overlapping. The red dots represent the original sample values. The black dots represent the bootstrap values. The gray areas represent the 95% bootstrapped confidence intervals. The bootstrapped confidence intervals were narrow, suggesting the accuracy of the results.

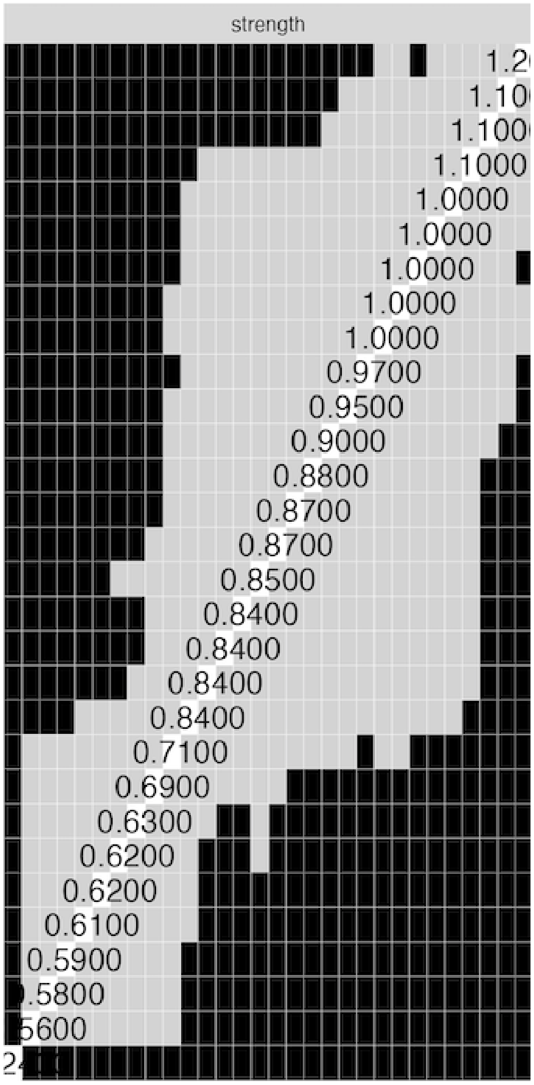


**Supplement Figure 3.:** Nonparametric bootstrapped centrality difference test at baseline (number of bootstraps = 1000). A bootstrapped difference test (α= 0.05) was used to determine whether the nodes were significantly different from each other regarding centrality measures. Each point on the x and y axes represents a network node. The gray boxes indicate that the two nodes do not significantly differ from each other. The black boxes indicate that the two nodes differ significantly from each other. The numbers on the diagonal represent the values of the centrality measure of the node.

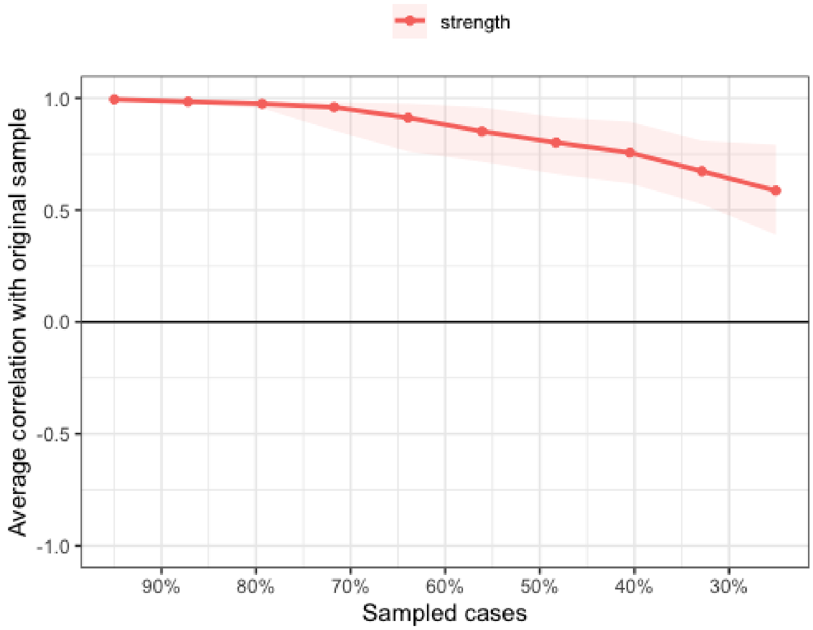


**Supplement Figure 4.:** Case-dropping bootstrapped procedure of strength at the two-year follow-up (number of bootstraps = 1000). The correlations of the centrality measure strength between the original sample and those from the subsamples with an increasingly higher percentage of dropout cases were calculated. The correlation stability coefficient (CS coefficient) represents the maximum proportion of cases that can be dropped to retain a correlation of at least 0.70 with the original strength in at least 95% of the samples. The 95% confidence interval of the correlation is indicated. The case-dropping bootstrap procedure showed that CS coefficient of node strength (CS(cor=0.7) = 0.52) remained high.

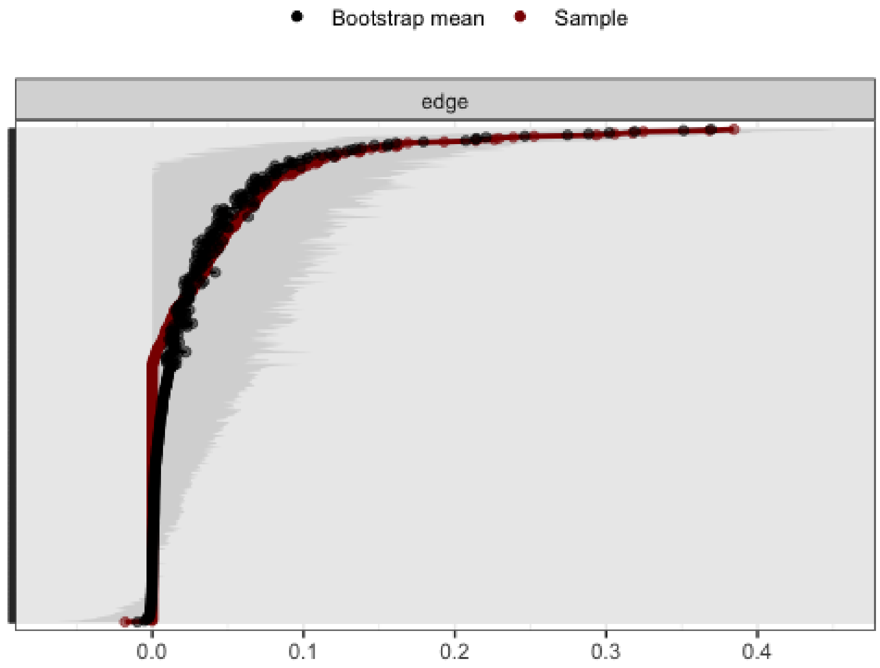


**Supplement Figure 5.:** Nonparametric bootstrapped procedure of edge weights at the two-year follow-up (number of bootstraps = 1000). Using the nonparametric bootstrapping procedure, the 95% bootstrapped confidence interval of the edge weights was determined around the regularized edge weights. Each horizontal line represents an edge. Y-axis labeling was omitted to avoid overlapping. The red dots represent the original sample values. The black dots represent bootstrap values. The gray areas represent the 95% bootstrapped confidence intervals. The bootstrapped confidence intervals were narrow, suggesting the accuracy of the results.

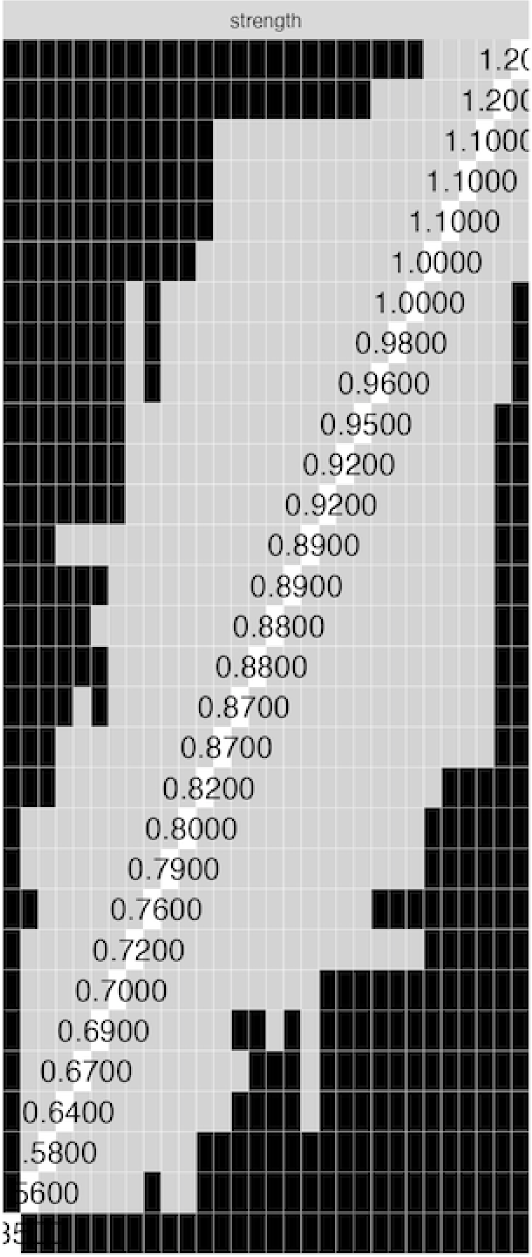


**Supplement Figure 6.:** Nonparametric bootstrapped centrality difference test at the two-year follow-up (number of bootstraps = 1000). A bootstrapped difference test (α= 0.05) was used to determine whether the nodes were significantly different from each other regarding centrality measures. Each point on the x and y axes represents a network node. The gray boxes indicate that the two nodes do not significantly differ from each other. The black boxes indicate that the two nodes differ significantly from each other. The numbers on the diagonal represent the values of the centrality measure of the node.

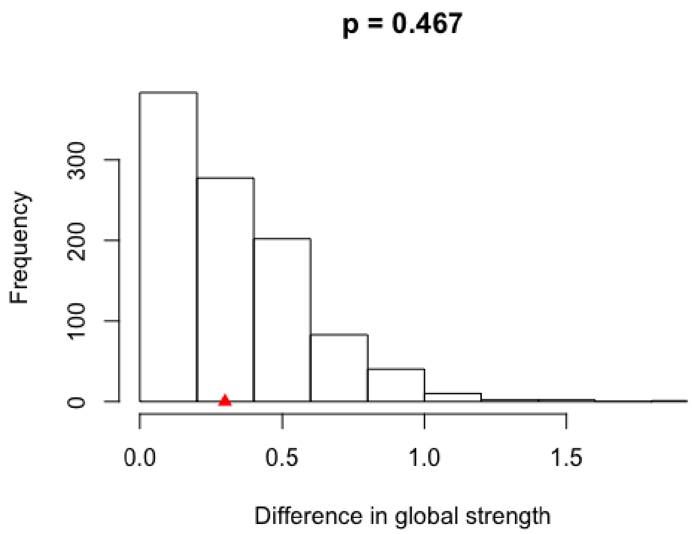


**Supplement Figure 7.:** Network comparison test of the differences in network global strength. A network comparison test was performed between the networks of the NMSS at baseline and the two-year follow-up.

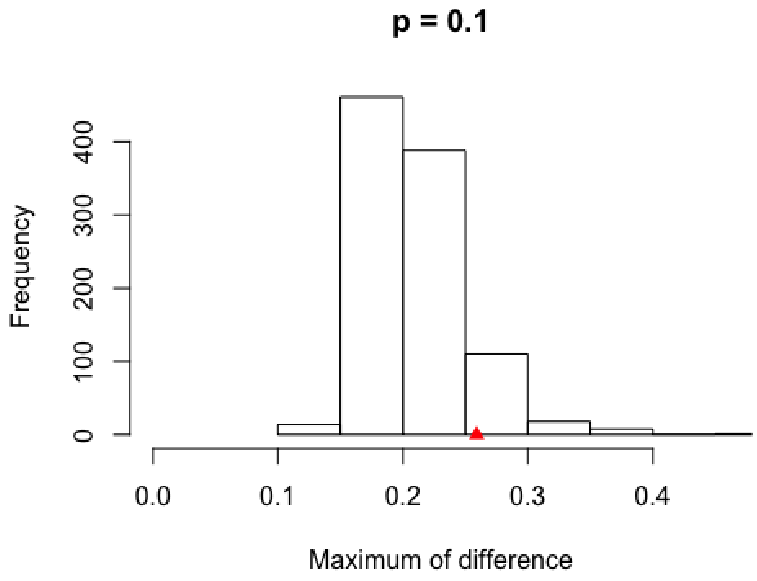


**Supplement Figure 8.:** Network comparison test of the differences in network structure. A network comparison test was performed between the networks of the NMSS at baseline and the two-year follow-up.
